# Reliability, convergent validity and factor structure of the DASS-21 in a sample of Vietnamese adolescents

**DOI:** 10.1371/journal.pone.0180557

**Published:** 2017-07-19

**Authors:** Minh Thi Hong Le, Thach Duc Tran, Sara Holton, Huong Thanh Nguyen, Rory Wolfe, Jane Fisher

**Affiliations:** 1 The Jean Hailes Research Unit, School of Public Health and Preventive Medicine, Monash University, Melbourne, Victoria, Australia; 2 Faculty of Social Sciences, Behaviours and Health Education, Hanoi School of Public Health, Hanoi, Vietnam; 3 Department of Epidemiology and Preventive Medicine, School of Public Health and Preventive Medicine, Monash University, Melbourne, Victoria, Australia; Iranian Institute for Health Sciences Research, ISLAMIC REPUBLIC OF IRAN

## Abstract

**Objectives:**

To assess the internal consistency, latent structure and convergent validity of the Depression, Anxiety and Stress Scale-21 (DASS-21) among adolescents in Vietnam.

**Method:**

An anonymous, self-completed questionnaire was conducted among 1,745 high school students in Hanoi, Vietnam between October, 2013 and January, 2014. Confirmatory factor analyses were performed to assess the latent structure of the DASS-21. Factorial invariance between girls and boys was examined. Cronbach alphas and correlation coefficients between DASS-21 factor scores and the domain scores of the Duke Health Profile Adolescent Vietnamese validated version (ADHP-V) were calculated to assess DASS-21 internal consistency and convergent validity.

**Results:**

A total of 1,606/ 1,745 (92.6%) students returned the questionnaire. Of those, 1,387 students provided complete DASS-21 data. The scale demonstrated adequate internal consistency (Cronbach α: 0.761 to 0.906). A four-factor model showed the best fit to the data. Items loaded significantly on a common general distress factor, the depression, and the anxiety factors, but few on the stress factor (p<0.05). DASS-21 convergent validity was confirmed with moderate correlation coefficients (-0.47 to -0.66) between its factor scores and the ADHP-V mental health related domains.

**Conclusions:**

The DASS-21 is reliable and suitable for use to assess symptoms of common mental health problems, especially depression and anxiety among Vietnamese adolescents. However, its ability in detecting stress among these adolescents may be limited. Further research is warrant to explore these results.

## Introduction

It is reported that mental disorders, including depression, are among the top 20 leading causes of disability worldwide [1]. All over the globe, about 400 million people are affected by depression [2] and around 10–20% of the world’s children and adolescents suffer from mental disorders [3]. These disorders may result in impaired capacity to study and work and even suicide among the sufferers if untreated [2]. Early detection of these conditions is therefore of great importance.

One of the commonly-used scales for detection of mental health problems is the Depression, Anxiety and Stress Scale [4]. Since its introduction in 1995, the DASS and its short-form—the DASS-21—have been widely used to assess depression, anxiety and stress among adults. Analyses of data among adults using this measure yield consistent results about its psychometric properties [4–7]. It has been shown to be reliable and valid with a three-factor structure [7,8]. The reliability and validity of both the DASS and the DASS-21 have been replicated among clinical [7,8], as well as non-clinical adult samples [6,9–11]. The same three-factor structure has been seen when the measure is used among diverse cultural and ethnic groups [12,13]. The DASS-21 has also been shown to have strong concurrent validity with other measures of depression, anxiety and stress including the Beck Depression Inventory, the Beck Anxiety Inventory and the State-Trait Anxiety Inventory Trait [14].

### The psychometric properties of DASS-21 among adolescents

Apart from consistent findings about the high internal consistency of the DASS-21 among adolescents [15–17], there are inconsistent findings about the factor structure of the DASS-21 when used among them, ranging from one- [17], two- [18] and three- [19] [15] to four- [15] factor structures.

As the DASS and DASS-21 were developed in Australia, their application among adolescents who live in other countries, speak different languages and have different cultures and context, requires careful consideration. There has been evidence that an instrument validated in one setting may not be valid when applied in another due to differences in language expression, cultural and social characteristics, development, values and beliefs [20].

Evidence about the reliability, validity and latent structures of the DASS and DASS-21 among adolescents in low and low-middle income countries is very limited. Assessing the factor structure of the DASS-21 among Malaysian adolescents aged 13–14 years, Hashim et al [21] found similar results to Patrick et al’s Australian study [17] which supported a one-Negative-Affect-factor solution.

### The use of DASS-21 in Vietnam

In Vietnam, the DASS-21 has been validated among women during the perinatal period [22]. Using a gold standard validation method in which the scores from this instrument were compared with diagnostic interviews, Tran et al [22] found that the scale failed to differentiate depressed women from anxious ones and that exploratory factor analysis yielded only one “emotional state” factor, instead of the original three factors. However, the internal consistency reliability of the scale was acceptable to high, with a Cronbach Alpha of 0.72 for the Depression, 0.77 for the Anxiety, 0.70 for the Stress subscale and 0.88 for the overall scale. The authors recommended the use of the scale in rural Vietnam as a reliable and sensitive screening tool for common mental disorders, but not for depression or anxiety separately.

This Vietnamese validated version of the DASS-21 was also administered among undergraduate students from Central Vietnam to assess the effectiveness of self-regulated learning strategies on reducing symptoms of depression [23]. The internal consistency of the scale was further confirmed with Cronbach Alpha’s of 0.81, 0.75 and 0.78, respectively, for the three subscales [23]. However, the factor structure of the DASS-21 was not examined in this study.

The use of the DASS-21 among adolescents in Vietnam has been limited to one study [23]; to date there has been no research examining its factor structure and convergent validity among adolescents. Without evidence about the psychometric characteristics of the instrument, the application of the original subscales scoring for adults to adolescents may result in misleading conclusions.

The aim of this paper was to examine the internal consistency, latent structure and convergent validity of the DASS-21 Vietnamese validated version (DASS-21-V) [22] among Vietnamese adolescents. Convergent validity of the DASS-21-V was assessed by comparing it to the Duke Health Profile Adolescent Vietnamese validated version (ADHP-V)—a health-related quality of life measure which has been validated among this population subgroup. We hypothesised that the factor scores of the DASS-21-V would show negative correlations with the ADHP-V domain scores and the largest correlations would be with the mental health domains (including mental health, anxiety and depression).

## Method

### Participants

This research uses data from a larger study; the aims of which were to investigate exposure to poly-victimisation and its associations with health and wellbeing of adolescents in Vietnam. Details of the study have been reported elsewhere [24]. In brief, the study was conducted during October, 2013—January, 2014 in Hanoi, Vietnam. This is the capital city of Vietnam with a population of 6.9 million people [25] and inclusion of urban as well as rural districts. Eighty six percent of the Vietnamese population belongs to the Kinh ethnic group [26]. Children and adolescents account for a third of the population [27] and almost all (97%) of 15-24-year-old adolescents and young adults are able to read and write [28].

Participants were students studying in public and private high schools and centres for continuing education in rural and urban areas of Hanoi, Vietnam. Ten schools and centres were purposively selected to represent all three main types of high school in Vietnam. None of the schools and centres invited refused to participate. Four to six classes were randomly selected from each school or centre and all students in these classes were invited to participate.

### Materials

#### Study specific questions

Participants’ socio-demographic characteristics, including age, sex, ethnicity, residential area and school type were assessed using study-specific questions. Socioeconomic status was determined using the World Bank’s assessment of the acquisition of 12 household items [29].

#### Depression, anxiety and stress scale– 21

The DASS-21 is the shortened version of the DASS developed by Lovibond and Lovibond [5] to assess symptoms of depression, anxiety and stress among adults. In the DASS-21 [5] the respondent is asked to think about their experiences in the past seven days and to judge how each statement applied to them. There are 21 items in this scale with four response options: 0 “Did not apply to me at all–Never”, 1 “Applied to me to some degree, or some of the time–Sometimes”, 2 “Applied to me to a considerable degree, or a good part of time–Often” to 3 “Applied to me very much, or most of the time–Almost always”. Scores on three subscales naming DASS-21-Depression (DASS-21-D), DASS-21-Anxiety (DASS-21-A) and Stress (DASS-21-S) can then be calculated [5]. There are seven items in each of the subscales; the score of which ranges from 0 to 21 [5]. The DASS-21 has been validated for use among Vietnamese samples [22]. The original English version [5] and its Vietnamese validated version [22] are described in S1 File.

#### The Vietnamese validated adolescent version of the Duke Health Profile (ADHP-V)

The Duke Health Profile (DHP) is a 17-item measure for health-related quality of life (HRQoL) [30]. The respondents were asked about their experiences in different timeframes including today or the previous week. It allows assessment of ten HRQoL domains, including physical, mental, general, social and perceived health and self-esteem, pain, disability, anxiety and depression [30]. The DHP was adapted for use among adolescents [31] and this version of the DHP was validated for use among Vietnamese adolescents [32]. The ADPH-V has been shown to have high internal consistency, acceptable reproducibility and confirmed construct validity [32]. Ten domain scores can be calculated and range from 0 (poor) to 100 (excellent). Higher scores indicate better HRQoL [32].

### Procedure

Students and their legal guardians received information packages about the study several days before the administration of the survey. Parental opt-out consent, in which parents were asked to complete a withdrawal form if they did not wish their child to participate, was sought for students less than 18 years of age. The survey was administered during a normal 45-minute-class session. An anonymous, self-complete questionnaire and a blank envelope were distributed to students on the survey day. Students’ consent to participate was implied by their completion of the questionnaire. Those whose parents completed the withdrawal forms were asked to put the blank questionnaire and the withdrawal form into the envelope and stay quietly in the classroom. Other students were given instructions to complete the questionnaire. All questionnaires were put in separate envelopes and handed back to the researchers at the end of the class session.

### Ethics approval

Ethics approval for the conduct of the study was given by the Institutional Review Board of the Hanoi School of Public Health (Application number 013-148/DD-YTCC) and the Human Research Ethics Committee of Monash University (project number CF13/1762-2013000897). Letters of approval were sought from the Heads of all schools and centres.

### Data analysis

Cronbach alpha statistics were calculated for the overall scale and the three subscales to assess reliability in terms of internal consistency. Confirmatory factor analyses (CFA), using structural equation modelling in Stata 12.0 [33], were performed to assess different latent structure models of the DASS-21-V. Maximum likelihood was the model estimation method used. Models examined were based on the results from previous research about factor structures of the DASS-21 among adolescents [15,18,19,21,22,34]. Specifically, we tested models with one, two, three and four factors. Model fit was assessed using likelihood ratio statistics, Root Mean Square Error of Approximation (RMSEA), Akaike’s Information Criterion (AIC), Bayesian Information Criterion (BIC), Comparative Fit Index (CFI) and Standardised Root Mean-squared Residual (SRMR). The model was considered to have good fit if the likelihood ratio p-value ≥ 0.05 [35], RMSEA ≤ 0.06 [15], CFI ≥ 0.90 [19] & SRMR ≤ 0.08 [36]. However, it should be noted that for large samples, a significant likelihood ratio p-value (<0.05) is likely to be obtained. Models with smaller AIC are said to have better fit than models with higher AIC [15]. Models with smaller BIC are also favoured than models with larger BIC [37]. A difference of more than 10 between BICs of two models is considered to show very strong evidence to favour the model with the smaller BIC [37].

To assess factor invariance, principal component factor analyses were run separately for girls and boys, and the numbers of principal factors compared between two groups. Various confirmatory factor analyses using the best-fit model, in which factor loadings and/or intercepts were free and then constrained, were also performed separately for girls and boys, and the models compared to assess factorial invariance.

Factor scores from the best-fit model were calculated and the correlations between these factor scores and the ten domain scores of the ADHP-V were examined. These correlations were used to assess the convergent validity of the DASS-21-V among this sample.

## Results

A total of 1,616/ 1,745 (92.6%) students returned a completed questionnaire. Out of those who did not participate, 120 were absent on the survey day; seven students and two parents refused participation. Complete responses for the DASS-21-V were available for 1,387 students (85.8%) and the analysis was restricted to these individuals. Socio-demographic characteristics of the analysis sample are presented in Table 1.

**Table 1:** Socio-demographic characteristics of 1,387 high school students with complete data for the DASS-21.

Variable	Sample Statistics
*Age (mean (SD))*	16.5 (1.0)
*Gender (n (%))*^*a*^	
Female	641 (46.3)
Male	744 (53.7)
*Ethnicity (n(%))*^*a*^	
Kinh	1,373 (99.6)
Others	4 (0.3)
Don’t know	2 (0.1)
*Residential area (n (%))*	
Rural	670 (48.3)
Urban	717 (51.7)
*Socioeconomic status (n (%))*^*a*^	
Poorest 25%	328 (24.8)
26–50%	329 (24.9)
51–75%	472 (35.7)
Richest 25%	192 (14.5)
*School type (n (%))*	
Public school	607 (43.8)
Private school	529 (38.1)
Centre for continuing education	251 (18.1)

SD: Standard deviation; n: number

^a^ Total N is different from 1,387 due to missing data.

### Summary statistics and reliability of the DASS-21-V

Summary statistics of the DASS-21-V for the whole sample, and separately for male and female students, are presented in Table 2. Standardised scores, which allow comparison with the DASS, are presented in Table 3. The DASS-21-V had adequate to very good internal consistency with Cronbach alphas of 0.906 for the overall scale, 0.835 for the Depression subscale, 0.737 for the Anxiety subscale and 0.761 for the Stress subscale.

**Table 2:** Summary statistics for the DASS-21-V among 1,387 high school students in Vietnam.

	Total sample				Males				Females			
	M	IQR	*M*	SD	M	IQR	*M*	SD	M	IQR	*M*	SD
Depression (0–21)^a^^**^	3	6	4.4	4.3	2	5	3.9	4.3	4	6	5.1	4.4
Anxiety (0–21)^a^^**^	4	5	4.7	3.9	3	5	4.2	3.7	5	6	5.4	3.9
Stress (0–21)^a^^**^	6	6	6.2	4.2	5	6	5.6	4.3	7	6	7.0	4.0
Total (0–63)^a^^**^	13	14	15.4	11.2	11	13	13.6	11.1	16	15	17.5	10.9

IQR: Inter Quartile Range; M: Median; *M*: Mean; SD: Standard Deviation

^a^ Range of scores is in brackets

** p<0.001 for between-group comparison between males and females.

**Table 3:** Standardised scores^a^ of the DASS-21-V among 1,387 high school students in Vietnam.

	Total sample		Males		Females	
	*M*	SD	*M*	SD	*M*	SD
Depression (0–42)^b^^**^	8.9	8.7	7.8	8.5	10.2	8.7
Anxiety (0–42)^b^^**^	9.5	7.8	8.4	7.5	10.8	7.9
Stress (0–42)^b^^**^	12.5	8.5	11.2	8.6	14.0	8.1
Total (0–126)^b^^**^	30.8	22.3	27.3	22.2	34.9	21.8

*M*: Mean; SD: Standard Deviation

^a^ Raw scores of the DASS-21-V were multiplied by two to create the standardised scores

^b^ Range of scores is in brackets

** p<0.001 for between-group comparison between males and females.

### Factor structures of the DASS-21-V

At first, we tested the original three-factor structure (3F-model 1), containing depression, anxiety and stress, as recorded among adult samples [6,15,18,19]. The three factors were allowed to correlate in this model. This model did not provide a satisfactory fit (Table 4). There were strong correlations between the three factors (standardised correlation coefficients between the DASS-21-D and DASS-21-A was 0.86; between the DASS-21-D and DASS-21-S 0.89 and between the DASS-21-A and DASS-21-S 0.93), suggesting indistinguishability of these factors. Modification indices were examined and we allowed for cross-loadings of items 9 and 13 on the DASS-21-S on the basis of substantial improvement of model fit for limited additional model complexity (3F-model 2). While improvements were seen in the chi-square statistic, the RMSEA and the AIC compared to the first model, overall there was still not a satisfactory fit.

**Table 4:** Model fit indices for confirmatory factor analyses of DASS-21 among 1,387 high school students in Vietnam.

Model	χ^2^	df	p-value	RMSEA	AIC	BIC	CFI	SRMR
**3F model 1**	1328	186	<0.001	0.067	67524	67869	0.883	0.044
**3F model 2**	1193	184	<0.001	0.063	67393	67748	0.896	0.044
**3F model 3**	1348	186	<0.001	0.067	67543	67889	0.881	0.044
**3F model 4**	1178	190	<0.001	0.061	67365	67690	0.899	0.042
**3F model 5**	1178	186	<0.001	0.062	67373	67719	0.898	0.042
**2F model 6**	2171	189	<0.001	0.087	68357	68676	0.797	0.115
**4F model 7**	**860**	**165**	**<0.001**	**0.055**	**67097**	**67553**	**0.929**	**0.034**
**3F model 8**	Failed to converge							
**1F model 9**	1540	189	<0.001	0.072	67730	68060	0.861	0.047
**4F model 10**	Failed to converge							

χ^2^: likelihood ratio chi-square statistics, df: degree of freedom for the likelihood ratio test of the model versus saturated, RMSEA: Root mean square error of approximation, AIC: Akaike’s information criterion, BIC: Bayesian information criterion, CFI: Comparative Fit Index, SRMR: Standardised Root Mean Squared Residual.

In the next stage, we examined the tripartite solution suggested by Duffy et al [18] in which the three factors comprised a physiological arousal factor (items 2, 4, 7 and 19), a lack of positive affect factor (items 3, 10, 16 and 21) and a general distress factor (the remaining items) (3F-model 3). These three factors were also allowed to correlate. The results again show an unsatisfactory fit (Table 4). The three factors correlated strongly with each other: *r* = 0.70 for physiological arousal-lack of positive affect-correlation, 0.78 for physiological arousal-general distress-correlation and 0.91 for lack of positive affect-general distress correlation. Based on modification indices, we allowed item 17 (“not worth much as a person”) to load on to the lack of positive affect factor (3F-model 4). There was a minor improvement in model fit statistics compared to 3F-model 3, but it was still unsatisfactory. The factor loading of item 17 on the General Distress factor in this model was very low (4.2 x 10^−5^); this loading was then removed in the next model (3F-model 5). However the model still did not have adequate fit.

The very strong correlation between the lack of positive affect and general distress factors in 3F-model 4 shows evidence that these two factors may not be distinguishable. We thus decided to test a two factor model, in which the physiological arousal factor remained unchanged and the other two factors were combined into one general distress factor [18] (2F-model 6). This model performed worse than the three factor models 1–5 already considered.

Based on the observation from the 3F-model 1, where factor loadings were high, but there were very strong correlations between the three factors, we decided to examine a four-factor solution (4F-model 7) which had also been tested in Szábo’s study [15]. In this model, in addition to the Depression, Anxiety and Stress factors, a common General Distress (GD) factor, on which all items were allowed to load, was added. The inclusion of this factor was to examine whether Depression, Anxiety and Stress factors were in fact showing a common non-specific general distress while there were specific characteristics to them, which may not be captured by the common factor only. In this model (4F-model 7), the three factors of Depression, Anxiety and Stress were allowed to correlate. Despite large chi-square statistics and a significant p-value due to large sample size, the results for this model can be considered to show an adequate fit to the data [35]. The correlations between the GD and the Depression, Anxiety and Stress factors were *r* = 0.90; 0.80 and 0.79, respectively. The correlations between the DASS-21-D, DASS-21-A and DASS-21-S reduced considerably (*r* = 0.61 between depression-anxiety, 0.64 between depression-stress and 0.59 between stress and anxiety). These correlations provide strong evidence to support the theory that depression, anxiety and stress represent a general distress component while they also have distinct characteristics. Although this model was the most complex, considering it had the smallest AIC and BIC (a model fit statistic that incorporates a penalty for increased model complexity), it still can be said as showing the best fit to these data, compared to all other models examined.

Tully et al [19] found that the model fit indices improved significantly when the Stress factor was removed from the model, leaving only the Depression, Anxiety and GD factors. However, when we applied this model in our sample (3F-model 8) the model estimation procedure failed to converge. We also tested a one-factor model (1F-model 9) as found by Tran et al [22] among rural Vietnamese women. However, this model also resulted in a poor fit relative to the other models. A four-factor model, in which there was a second-order factor linking all the three Depression, Anxiety and Stress factors but with no direct item loading on this factor and no correlation between Depression, Anxiety and Stress factors (4F-model 10) was also tested. Convergence was not reached for this model.

#### Factor invariance

Results from the principal factor analyses conducted separately for girls and boys revealed four factors in both groups (see Table 5), which indicated configurational invariance. Confirmatory factor analyses using the best-fit model (4F-model 7) in which factor loadings and intercepts were not constrained and grouping according to sex was performed showed that factor loadings and intercepts were automatically set to be equal across groups for all four factors. Another model in which factor loadings and intercepts were constrained to be equal across groups produced similar results to the previous model. Because results produced from the two models were exactly the same, it was not possible to assess whether there was metric invariance. Similar to previous publications which examined factor structures of the DASS-21 among adolescents [15–17] [18] [19], subsequent analyses were then performed on the sample as a whole.

**Table 5:** Number of factors of the DASS-21-V among girls and boys in a sample of 1,616 Vietnamese high school students.

Component	Female Eigenvalues	Male Eigenvalues
**Component 1**	7.03664	7.7051
**Component 2**	1.37416	1.39909
**Component 3**	1.20366	1.16565
**Component 4**	1.11314	1.03738

#### Factor loadings of the best-fit model

**Fig 1:**
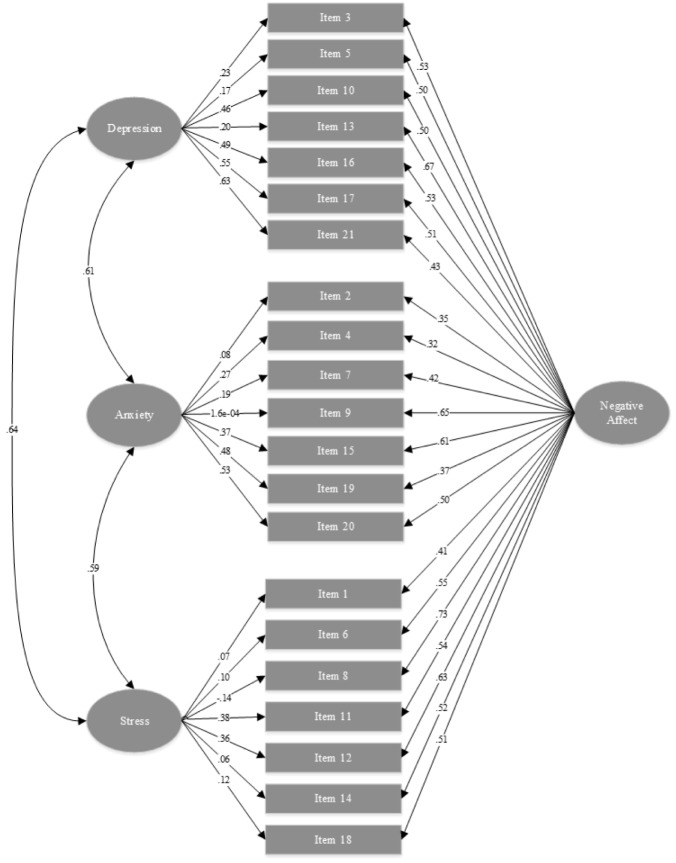
Factor loadings of the DASS-21-V among a sample of adolescents in Vietnam (errors for all items were removed for simplicity of the figure).

Details of the factor loadings for the 4F-model 7, which provided the best fit, are presented in Fig 1. Overall, all item loadings on the GD factor were significant and high, ranging from 0.32 to 0.73. Item loadings on the Depression factor were also significant but not as high, ranging from 0.17 to 0.63. For the Anxiety factor, six item loadings were significant, except for item 9 (panic and make fool of self); ranging from 0.081 to 0.53. The Stress factor has the least number of significant items of only two (item 11 –agitated and item 12 –difficult to relax) with factor loadings of 0.38 and 0.36, respectively.

### Convergent validity

Correlations between scores of the four factors (General Distress, Depression, Anxiety and Stress) retrieved from the 4F-model 7 and ten domain scores of the ADHP-V are presented in Table 6. As hypothesised, all four factors had negative correlations with the domain scores of the ADHP-V. The General Distress, Depression, Anxiety and Stress factors correlated strongest with the general health, anxiety, depression and mental health domains of the ADHP-V. Since the score of the general health domain was calculated as the average of the physical, mental and social health domain scores, this finding is not surprising. The lowest correlations were with the disability domain (which indicated occurrence of any sickness or injury or health problem to the adolescents).

**Table 6:** Correlation coefficients between four factor scores of the depression, anxiety and stress scale Vietnamese validated version and ten domain scores of the Vietnamese validated adolescent version of the Duke Health Profile.

	DASS-21-V							
	General Distress		Depression		Anxiety		Stress	
	Corr coef	P-value	Corr coef	P-value	Corr coef	P-value	Corr coef	P-value
ADHP-V Mental health	-0.63	<0.001	-0.58	<0.001	-0.48	<0.001	-0.47	<0.001
ADHP-V Anxiety	-0.64	<0.001	-0.57	<0.001	-0.50	<0.001	-0.50	<0.001
ADHP-V Depression	-0.64	<0.001	-0.58	<0.001	-0.51	<0.001	-0.48	<0.001
ADHP-V Self-esteem	-0.52	<0.001	-0.47	<0.001	-0.35	<0.001	-0.39	<0.001
ADHP-V Pain	-0.37	<0.001	-0.30	<0.001	-0.33	<0.001	-0.28	<0.001
ADHP-V Disability	-0.10	<0.001	-0.07	<0.01	-0.14	<0.001	-0.11	<0.001
ADHP-V Physical health	-0.50	<0.001	-0.43	<0.001	-0.45	<0.001	-0.39	<0.001
ADHP-V Social health	-0.31	<0.001	-0.32	<0.001	-0.21	<0.001	-0.24	<0.001
ADHP-V Perceived health	-0.21	<0.001	-0.19	<0.001	-0.19	<0.001	-0.14	<0.001
ADHP-V General health	-0.66	<0.001	-0.61	<0.001	-0.52	<0.001	-0.50	<0.001

Corr coef: Correlation coefficient.

## Discussion

In this study, the factor structure and evidence about the convergent validity of the DASS-21 Vietnamese validated version among a large sample of Vietnamese adolescents is presented for the first time. The results demonstrate that the DASS-21-V is reliable and shows acceptable convergent validity among these adolescents. We also found that a four-factor latent structure (4F model 7), representing general distress, depression, anxiety and stress factors, best fit the data among these students. The results are robust with a systematically recruited, large sample, participants representing students from three main types of high schools and institutions in Vietnam, a high response rate and a low level of incomplete data.

Similar to previous research, we have found that the factor structure of the DASS-21 among adolescents is different to that among adults [15,18,19,21,22,34]. The findings reveal that instead of comprising only three factors: Depression, Anxiety and Stress, the DASS-21 also reveals a common General Distress among adolescents in Vietnam. This is consistent with the results from Szabo’s study among a sample of Australian adolescents [15]. The author also found that this 4-factor-model provided the best fit to their data compared to other models [15]. As suggested by Szabó [15], these results show that the underlying common factor of General Distress may have explained the high correlations among the three subscales of the DASS-21 among adolescents [15].

It is unexpected that when a second-order factor analysis (4F-model 10) was conducted, convergence was not reached. This result again suggests that among these Vietnamese adolescents, the conventional DASS-21 factors of Depression, Anxiety and Stress did not perform in the same way as they do among adults. It might have been that the shared component among these three factors may not be large enough to represent the variances among them and to be retrieved as a second-order factor.

Our findings that the four-factor model (4F model 7) was more superior than the one factor model (1F model 9) provide important evidence that among these Vietnamese adolescents, the DASS-21 showed not only a common non-specific general distress factor, but also specific, distinguishable components to each of the subscales of Depression, Anxiety and Stress. This result supports previous research which has indicated the inter-related, but distinct characteristics between depression and anxiety and between stress and negative affect [4,9,14,16,38].

This study adds important information about factor invariance of the DASS-21 among adolescent boys and girls. We have been able to establish configurational invariance, providing supporting evidence of equal number of latent factors of the DASS-21-V between girls and boys in this sample. However, we were not able to conclude whether each item were similarly understood by girls and boys. Future research examining similarities or differences in terms of factor loadings and intercepts of the DASS-21-V between the two sexes is thus needed.

Further examinations of the item loadings of the four-factor model show that the seven items of the DASS-21-D generally loaded well on this factor in addition to the common General Distress factor. This provides evidence that among these Vietnamese adolescents, symptoms of depression, which in this case, were characterised by items showing a lack of positive affect and loss of interest or pleasure, may be similar to those among adults and the use of the DASS-21 may be able to detect these. Item loadings of those belonging to the DASS-21-A were not as good, especially for item 2 (dryness of the mouth) and item 9 (panic and make fool of self). These items were loaded primarily on the common General Distress factor. Items loadings for the other items (item 4, 7, 15, 19 & 20) were low to moderate (0.19–0.53), but significant, suggesting that symptoms of physiological arousal may be indicators of anxiety among Vietnamese adolescents. Symptoms of ‘dryness of the mouth’ or ‘feeling panic” and ‘making a fool of oneself’, however, may not represent symptoms of anxiety among this sample. Further research to identify other items which are more suitable and specific to anxiety among Vietnamese adolescents may be helpful.

For the DASS-21-S, item loadings were only significant for item 11 (Agitated) and 12 (difficult to relax). For all other items, the loadings were very small and even negative (item 8 –using a lot of nervous energy). The Vietnamese translation of item 1 (‘hard to wind down’) was not able to capture the original English meaning of the item, but instead, was read as “hard to control oneself”. Similar problems were identified with the translation of item 18 (‘rather touchy’). It was only stated as “I felt that I was sensitive” and failed to convey the meaning of being “easily moved to anger” or “highly sensitive in temper or disposition” (Oxford dictionary). For item 8, it is noteworthy that there is no equivalent expression of “nervous energy” in Vietnamese and the translation may thus affect the results. These findings again replicated Szabo’s observation that the DASS-21 items used to assess “Tension/ Stress” among adults may not be adequate to enable assessment of this construct among adolescents or that adolescents’ emotional development may not have yet reached a state similar to that of adults [15].

Our results, however, were different from those reported in other research among Australian sample of children and adolescents [17–19] and Malaysian adolescents [21] who found one-factor, two-factor or three-factor structure models. Differences in the participants’ age between our sample (15–18) and the other samples (11–18) may explain these results. Adolescents in our study were older and their development may thus have been different from those of the other samples. These differences may also be accounted for by the different economic and social characteristics between Vietnam and the other two countries, resulting in different levels of emotional awareness and recognition among the adolescents. Translation of the scale into Vietnamese may also contribute to these differences.

Compared to the results from Tran et al’s study [22], the findings in this study also differ. Tran et al [22] administered the DASS-21-V among a sample of rural Vietnamese women during the perinatal period and concluded that the data supported a one-”Emotional-State”-factor model. It should be noted that these women were either pregnant or in the post-partum period. Their characteristics may have been very much different from those of the adolescents in this study. The factor structure of the DASS-21 when applied among perinatal women therefore appears to be different from that among adolescents.

In this study, convergent validity of the DASS-21-V among Vietnamese adolescents was also confirmed. The four factors of the DASS-21-V showed strongest correlations with those domains of the ADHP-V which were expected to measure the same constructs, while they showed weak to very weak correlations with those domains which measured different constructs. Specifically, moderate negative correlation coefficients (ranging from -0.64 to -0.47) were observed between the four factors of General Distress, Depression, Anxiety and Stress of the DASS-21-V and the mental health, anxiety and depression domain scores of the ADHP-V; weak to very weak negative correlations (ranging from -0.35 to -0.04) were recorded between the four factors of the DASS-21-V and the social and perceived health and disability domains of the ADHP-V.

There are some limitations in this study, which should be considered in interpreting the results. The schools and centres were not randomly selected. Adolescents not in school were not included. The participants resided in the capital city of Vietnam, the economic and socio-demographic development of which may be more advanced than other regions and the emotional awareness and recognition of adolescents may be different from those living elsewhere. The results may thus not be generalisable to out-of-school adolescents or those living in remote areas.

## Conclusions

Overall, the results from this study provide evidence that the DASS-21-V is suitable for use as a screening tool for symptoms of common mental health problems, especially Depression and Anxiety among adolescents in Vietnam. The study also extends knowledge on the reliability and convergent validity of the DASS-21-V among Vietnamese people. We recommend the use of a total DASS-21 score to represent symptoms of general distress when applied among Vietnamese adolescents. The use of subscale scores of Depression and Anxiety among them is also acceptable. Nevertheless, the DASS-21-V to detect symptoms of stress among Vietnamese adolescents should be used with caution. Future research in which the DASS-21-V is compared against a standard diagnostic instrument for common mental disorders would be helpful in confirming its validity for use among Vietnamese adolescents. Revision of items or even substitution of new items specific to these constructs among adolescents should be considered in such research.

## Supporting information

S1 File The DASS-21 in English and Vietnamese. (DOCX)
