# Supplementary material for: Reliability, convergent validity and factor structure of the DASS-21 in a sample of Vietnamese adolescents
Source: PLoS One. 2017 Jul 19;12(7):e0180557. doi: 10.1371/journal.pone.0180557 (PMC5516980; doi:10.1371/journal.pone.0180557)
Supplement: S1 File — (DOCX) [file pone.0180557.s001.docx]

| Item | Original English | Vietnamese |
| --- | --- | --- |
| 1 | I found it hard to wind down | Tôi rất khó kiềm chế bản thân |
| 2 | I was aware of dryness of my mouth | Tôi thường hay khô miệng |
| 3 | I couldn’t seem to experience any positive feeling at all | Tôi dường như không có những cảm giác vui vẻ |
| 4 | I experienced breathing difficulty (eg, excessively rapid breathing, breathlessness in the absence of physical exertion) | Tôi thấy khó thở (thở nhanh, khó thở khi không làm việc mệt) |
| 5 | I found it difficult to work up the initiative to do things | Tôi thấy khó bắt tay vào một công việc nào đó |
| 6 | I tended to over-react to situations | Tôi thường có xu hướng phản ứng thái quá với sự việc |
| 7 | I experienced trembling (eg, in the hands) | Tôi cảm thấy mình hơi run (ví dụ như run tay) |
| 8 | I felt that I was using a lot of nervous energy | Tôi thấy mình suy nghĩ quá nhiều |
| 9 | I was worried about situations in which I might panic and make a fool of  myself | Tôi thường lo lắng về những tình huống mà tôi thấy hoảng sợ và bị mất mặt với người khác |
| 10 | I felt that I had nothing to look forward to | Tôi thấy mình chẳng có gì để mong đợi |
| 11 | I found myself getting agitated | Tôi thấy mình bồn chồn |
| 12 | I found it difficult to relax | Tôi cảm thấy khó thư giãn |
| 13 | I felt down-hearted and blue | Tôi cảm thấy buồn và chán |
| 14 | I was intolerant of anything that kept me from getting on with what I was  doing | Tôi không chịu nổi khi công việc đang làm bị ngăn cản |
| 15 | I felt I was close to panic | Tôi cảm thấy lo sợ |
| 16 | I was unable to become enthusiastic about anything | Tôi thấy mình mất hết hứng thú với mọi thứ |
| 17 | I felt I wasn’t worth much as a person | Tôi thấy mình là người vô dụng |
| 18 | I felt that I was rather touchy | Tôi cảm thấy mình rất nhạy cảm |
| 19 | I was aware of the action of my heart in the absence of physical exertion (eg,  sense of heart rate increase, heart missing a beat) | Tôi thấy tim mình đập nhanh, loạn nhịp khi không hoạt động mạnh |
| 20 | I felt scared without any good reason | Tôi thấy sợ hãi vô cớ |
| 21 | I felt that life was meaningless | Tôi thấy cuộc sống không còn ý nghĩa gì |

Source: Lovibond & Lovibond (1995) [5] and Tran et al 2013 [22]
